# Clinics at Tri-State Dental Meeting

**Published:** 1901-11-15

**Authors:** 


### CLINICS AT TRI-STATE DENTAL MEETING.

### NEW METAL FOR CAST PLATES.

BY DR. R. C. BROPHY, CHICAGO, ILLS.

This was a method of casting a lower denture using a new alloy of Dr. Brophy's own devising, also a new investment composition. The claim was that the metal had a bright white color which it retained, and that it was more dense than other cast metals. The metal cast well, but the investment used made a rough surface which required considerable work to polish. The inventor claims for the investment that it does not change form under the heat used in casting, therefore the plates fit better. The heat for casting was generated by a gasoline furnace of Dr. Brophy's invention, which was neat in appearance and a very effective method of securing heat for such purposes.

### CARMICHAEL SYSTEM FOR CROWN AND BRIDGE WORK.

BY DR. J. P. CARMICHAEL, MILWAUKEE, WIS.

A number of prepared specimens of the application of this system for attaching bridges and restoring contours were shown. He also demonstrated the attachment of a bridge which he had in process of construction, showing the preparation of the teeth and method of constructing the retainers. Essentially the system is to cut a groove axially on the mesial and distal surfaces which extends across the occlusal surfaces, making a continuous groove from the gum on the mesial to the gum on the distal over the occlusal. A plate of metal is then fitted to the lingual or palatel surface and into this groove. When properly done this makes a secure and firm anchorage for a bridge and the tooth has not been irreparably mutilated, and the labial face is free from the unsightly appearance of metal.

For porcelain bridge work the method is applicable as for gold work. It is the greatest value in building out and restoring the contour of the incisors, when badly decayed on the mesial and distal surfaces and when broken through to the occlusal edge. Such cases as usually demand cutting off the crown entirely or fitting with a box crown. By this process a much more aesthetic result may be secured and with probably greater safety to the tooth. There are also many other valuable uses to which the system may be applied, such as inlays, protecting caps for abraded teeth, etc.

The advantages claimed are, adaptation to any tooth, strength, no mutilation of the teeth, conserves the pulp, hygienic, and avoids an undue display of metal in the mouth.

### CONTOURING PLIERS.

BY DR. D. MILLER, CHICAGO, ILLS.

These contouring pliers were unique. The points of a pair of round-nosed pliers are fitted with matched die and counter in the form of rolls, which revolve on the points of the plier and against each other, thus creasing or contouring the band as the case may need, and dependent on what form of roll is used. The rolls are readily interchangeable and a considerable variety can in this way be obtained. For some purposes it is without doubt a valuable appliance, but the crowns which we saw contoured with it, were not properly done. The lateral contracts were at considerable distance from the occlusal surfaces, which would leave wedge-shape spaces or grooves into which food particles would lodge and force their way into the interdental spaces.

### ADAPTING A PAD OF GOLD TO THE CERVICAL BORDER WITH A MATRIX.

BY DR. F. W. STEPHAN, CHICAGO, ILLS.

The Doctor demonstrated his method of securing the adaptation of foil to the cervical border of mesial and distal cavities. He folds the foil upon itself several times until he secures a mat of any desirable thickness, which he cuts to such size as will pass between the proximating teeth and cover the outlines of the cavity. The matrix is then slipped down beside it and wedged until it forces the foil tight against the tooth. With a sharp instrument the mat of foil is then cut and folded into the cavity. This insures an adaptation to the margin over which it is folded and protects it from plugger contact. The gold filling is then built in as usual. The mat holds the matrix away from the margins, so that there are no close angles to fill. A very helpful suggestion.

### REMOVABLE BEAKS FOR EXTRACTING FORCEPS.

BY DR. C. V. VIGNES, NEW ORLEANS, LA.

The Doctor exhibited a pair of forcep handles to which might be added the beaks of any forcep desired. The instrument was ingeniously constructed, but we doubt its practicability. There is more danger, we imagine, of breaking the beaks or having them loosen than in an ordinary forcep, and it would not facilitate extraction to have to stop and change beaks in the midst of an operation which it is better should be done as expediously as possible.

The Doctor also demonstrated his method of constructing dummies and gold crowns for bridge work, which was described in the discussion of Dr. Young's paper.

### PARKER SHOT SWEDGING MACHINE.

BY DR. B. H. LEE, GRAND RAPIDS, MICH.

The value of the Parker swedging apparatus in crown work, as well as for plate work, is well known. Casts with deep undercuts are easily used by this method. Aluminum and thin metal may be swedged on plaster molds.

### SANDVIG'S SWEDGING APPARATUS.

BY DR. I. OTTESEN, CHICAGO, ILLS.

Dr. Sandvig claims that by this method all kinds of metal plates can be swedged in one-tenth the time consumed by any other method.

The die is made direct from the plaster impression, without even waiting for it to dry, and it can be used any number of times without danger of its changing form. The metal baseplate is swedged direct upon this die, using sand as a counter die.

The metal used is very low fusing and free from zinc, thereby removing the objectionable feature of the old methods of swedging, whereby the aluminum is contaminated with the zinc.

Dr. Sandvig has been using aluminum plates swedged in this manner for the past six years, and has had the greatest success. He claims that aluminum should be used more as a base-plate in the future, and that a metal plate can be made almost as quickly as it can be done in rubber.

### FILLING ROOT CANALS WITH GOLD FOIL.

BY DR. I. DOUGLAS, ROMEO, MICH.

The doctor demonstrated his method of filling root canals with gold foil. The work was succesfully done, but we can see no advantage it can have over gutta-percha or other materials more readily and just as effectively manipulated. The doctor also showed some tin fillings, which were remarkable for restoration of contour and close adaptation.

### A NEW MAT GOLD.

BY DR. J. B. VERNON, MEMPHIS, TENN.

Dr. Vernon exhibited and demonstrated the method of using a new preparation of gold for filling teeth. It resembles the various crystal golds on the market.

It is of a more even texture than de Trey's and not so shredded as White's Moss Fibre. It is claimed for it that it can be thoroughly condensed by hand pressure, and does not ball or pull away from the cavity walls or margins, and is about as easy to manipulate as an alloy. Special oval-faced pluggers are advocated for using it, so that it may be spread latterally against the walls. The claim is also made that it can thus be condensed until it is practically as solid as melted gold and with just as good edge strength. It is probably another variety of crystal gold precipitated by electrolysis, and we have no doubt it will find many uses, but will not take the place of foil where hard wear is required.

---

### CARIOUS TOOTH IN A DERMOID CYST.

---

BY DR. J. R. CALLAHAN, CINCINNATI, O.

---

This was a large dermoid cyst, which contained several teeth, one of which showed unmistakable evidence of dental caries. In the crown of a molar a cavity involving perhaps one-third of the substance of the tooth, was a brown soft substance resembling exactly the brown leathery decay often seen in molar teeth. The other teeth were unaffected.

---

### METALLIC GUARD FOR ENGINE DISKS AND WHEELS.

---

BY DR. F. F. HOYER, OWOSSO, MICH.

---

This automatic metal disk guard for protecting the buccal membranes and tongue from contact with engine disks and wheels, when grinding the tooth, separating or polishing fillings, etc., consists of a cap-like piece of metal, resembling a round tin cover, about three-fourths of an inch in diameter, with flange wide enough to protect the soft tissues of the mouth. One side of the metal flange is cut out so as to expose the running surface of the disk after the guard has been slipped over the end of hand-piece in position. This allows of grinding the tooth or filling, but the rim prevents contact of disk with tongue, cheek, or other soft tissues of the mouth.

The appliance is ingenious and effective, but seems to obscure, somewhat, the view of the tooth.

